# Factors Associated With Severe Mental Illnesses Newly Diagnosed in Perinatal Psychiatric Care: Findings From a Large Clinical Cohort

**DOI:** 10.1177/10401237261434895

**Published:** 2026-03-12

**Authors:** Alexandra Painchaud, Marie-Josée Poulin, Célia Matte-Gagné, Chantal Mérette

**Affiliations:** 1 Centre de Recherche CERVO, Québec City, QC, Canada; 2 École de Psychologie, Université Laval, Québec City, QC, Canada; 3 Institut Universitaire en Santé Mentale de Québec, Québec City, QC, Canada; 4 Département de Psychiatrie et Neurosciences, Université Laval, Québec City, QC, Canada; 5 Groupe de Recherche sur l’inadaptation Psychosociale chez l’enfant (GRIP), Québec City, QC, Canada; 6 Centre de Recherche Universitaire sur les Jeunes et les Familles (CRUJeF), Québec City, QC, Canada; 7 Centre de Recherche du CHU de Québec, Université Laval, Québec City, QC, Canada

**Keywords:** perinatal, mental disorders, comorbidity, risk factors

## Abstract

**Background:**

The perinatal period represents a phase of increased psychiatric vulnerability, yet the factors linked to severe mental illnesses (SMIs) diagnosed during pregnancy or postpartum remain unclear. This study sought to identify associated and predictive factors of depression, bipolar disorder, and schizophrenia-related disorders diagnosed during pregnancy vs postpartum, with or without psychiatric comorbidity, in women seeking psychiatric care.

**Methods:**

Using a retrospective cohort design, data from 927 women who accessed specialized perinatal psychiatric services in Quebec City (Canada) were analyzed. Poisson regression models examined psychiatric, sociodemographic, medical, and obstetric correlates of perinatal SMIs.

**Results:**

Distinct profiles emerged according to diagnosis timing, disorder type, and comorbidity. During pregnancy, associated factors included being single, absence of prior psychotropic use, musculoskeletal conditions, and cluster C personality disorders. Past postpartum psychosis predicted bipolar disorder diagnosed during pregnancy. In the postpartum period, key correlates included history of anxiety or (hypo)manic symptoms, somatic-related disorder, suicidal behavior, absence of previous psychiatric diagnosis, and no new diagnosis during pregnancy. Foreign nativity and lack of psychotropic treatment were linked to depression across both periods.

**Conclusions:**

Results highlight period-specific and shared factors underlying perinatal SMIs. Their identification may improve early detection and targeted interventions for maternal mental health.

## Introduction

The perinatal period is a time of heightened vulnerability to psychiatric disorders, affecting over one in five women.^1-6^ We recently characterized retrospectively the psychiatric trajectories of help-seeking women, spanning from lifetime psychiatric history to the postpartum period, and identified a broad range of new diagnoses emerging during pregnancy or postpartum, encompassing more than 30 distinct diagnoses.^7^ This heterogeneity is compounded by diverse perinatal-diagnosed psychiatric comorbidities, observed in up to one third of women during pregnancy or postpartum, reflecting dynamic, multifaceted clinical trajectories and underscoring the complexity of these clinical pathways. A deeper understanding of common and specific factors associated with the diagnosis of severe mental illnesses (SMIs) – including depression, bipolar disorder, and schizophrenia-related disorders – along with their comorbidities, would help delineate this complexity.

The perinatal period involves complex physiological changes that may increase psychiatric vulnerability.^8,9^ Pregnancy is characterized by elevated levels of reproductive and stress hormones, which rapidly decline following delivery,^10^ suggesting different psychiatric pathophysiological processes based on the timing of diagnosis (pregnancy vs postpartum).^4,11^ Perinatal psychiatric disorders likely stem from multifactorial etiologies – biological, psychosocial and environmental – many of which remain poorly understood.^6^ Identifying specific factors associated with each period could clarify these mechanisms, as their influence may differ before and after childbirth.^12^

Most studies have focused on factors associated with a specific psychiatric disorder, comparing affected women to population-based samples. For instance, a past psychiatric history, particularly depression, is a well-established factor associated with depression during pregnancy (ie, prenatal depression; odds ratio (OR) = 3.17),^13,14^ with higher risk among those discontinuing antidepressants during pregnancy.^15^ Other associated factors include: premenstrual dysphoric disorder, personality traits, family psychiatric history, unplanned pregnancies, obstetric complications, and social factors such as being single/divorced, or from a minority ethnic group.^13,14,16-20^ Whereas age and parity have also been implicated, studies conflict on whether younger or older age, and primiparity or multiparity increase the risk of prenatal depression.^13^ Notably, many of these factors are also linked to depression in the general population.^14^ In contrast, little is known about the factors associated with the diagnosis of bipolar or schizophrenia-related disorders during pregnancy.^21^

Factors associated with postpartum depression (up to one year after childbirth) are well documented and include: a history of psychiatric disorders before or during pregnancy – particularly depression, premenstrual dysphoric, obsessive-compulsive, anxiety, bipolar or post-traumatic stress disorders – with ORs ranging from 2.05 to 22.33.^10,20,22-26^ Subclinical psychiatric symptoms during pregnancy also increase postpartum depression risk.^27^ Other factors, such as marital and migration status, have modest effects.^28,29^ The association between maternal age and postpartum depression is inconsistent, with both younger and older age identified as associated factors across studies, as reviewed by Norhayati et al (2015).^29^ Additionally, primiparity, unplanned or unwanted pregnancies, and obstetric complications modestly increased risk.^17,28,30-35^

Little is known about the factors associated with postpartum diagnoses of bipolar or schizophrenia-related disorders, although several studies have examined perinatal relapse in women with preexisting conditions.^21,36-43^

The present study investigated help-seeking women who received perinatal psychiatric care. We focused on SMIs newly diagnosed during pregnancy or postpartum, aiming to identify sociodemographic and clinical factors that preceded these diagnoses, to improve understanding of perinatal psychiatric care and support early detection.

## Method

### Participants and Design

Our cohort study included women who sought specialized care at a perinatal psychiatry clinic in Quebec City (Canada) between its opening, in 2004, and 2020. This highly specialized care clinic accepts referrals from any physician, including family doctors, obstetricians/gynecologists, and psychiatrists, as well as from midwives, for the assessment and management of moderate to severe mental health conditions during the preconception period, pregnancy, and up to 6 months postpartum. Women with at least one consultation during the study period were included. Those in preconception who never became pregnant were excluded (*n* = 37), yielding a final sample of 927 out of 964 patients. Sociodemographic and clinical data are detailed in Painchaud et al (2025).^7^ Postpartum care data were available for 772 women. Data were systematically collected in 2019-2020 through a medical record review conducted by a psychology PhD student, under the supervision of the psychiatrist-in-charge, using a grid specifically designed for the present project and inspired by the work of Aumais et al (2025) and Kattan et al (2020), a prospective, multisite clinical databank in Quebec.^44,45^ Ethical approval was obtained from the Neuroscience and Mental Health Research Ethics Committee of the *Centre intégré universitaire de santé et de services sociaux de la Capitale-Nationale* (CIUSSS-CN; project # 2020-1851). The request for access to patient medical records for research purposes was granted.

### Measures

#### Outcomes: Prenatal and Postpartum Diagnoses

Diagnoses were extracted from the women’s medical record which were all based on semi-structured interviews (modified SCID) conducted by the clinic’s psychiatrist-in-charge. Prenatal SMI was defined as a new case of depression, bipolar disorder (type I, type II, atypical, unspecified or cyclothymic), or schizophrenia-related disorders (schizophrenia, schizoaffective or substance-induced psychotic disorders) diagnosed during pregnancy. Postpartum SMI was similarly defined as a new diagnosis made within one year following delivery. All other psychiatric diagnoses were included in the broader outcome “any mental illness” (any-MI), covering up to 36 disorders. Prenatal and postpartum new disorders thus defined were used as the study outcomes. When a SMI was diagnosed with one of the any-MI condition within a same perinatal period, then the outcome became “SMI with comorbidity”. Each outcome had to be observed in ≥5 women to be part of the study.

#### Potential Associated Factors

Sociodemographic, medical and obstetric factors preceding the outcomes were extracted from medical records, including age at childbirth, nativity, marital status, medical history, parity, planned/wanted pregnancy, and current or previous obstetric complications. Past psychiatric history, psychiatric hospitalization, psychotropic use, and family psychiatric history, documented in the medical records, were also considered as potential associated factors. Additionally, outcomes during pregnancy were examined as potential factors associated with postpartum outcomes. Only factors observed in at least 5 women were included in the analysis (see Tables S1-S4 in the appendix for retained factors).

## Statistics

Statistical analyses were conducted using SAS/STAT software version 9.4 (SAS Institute Inc., Cary, NC, USA). We identified factors associated with our outcomes using Poisson regression models with robust error variances. Given that maternal age was often associated with a factor and/or the outcome, each model was adjusted for maternal age at childbirth to account for potential confounding and covariate effects. This approach, based on the Modified Poisson Regression method by Zou (2004),^46^ was preferred over log-binomial regressions to avoid convergence issues commonly encountered with the latter. Analyses were performed using the GENMOD procedure in SAS, specifying a log link function and a Poisson distribution (link = log, dist = poisson).

We assessed the association between a given factor, in a specific period, and an outcome in a subsequent perinatal period by calculating the proportion of women with the outcome among those exposed to the factor of interest over that proportion among those unexposed. This prospective approach yields a relative risk as a measure of the strength of the association, rather than an odds ratio. The associations thus detected between factors and outcomes across the perinatal period will help explain why some trajectories were more likely than others among those revealed in Painchaud et al (2025).^7^ Univariate regression analyses were first performed considering one factor at a time for each outcome. We reported one-sided *p*-values and relative risks adjusted for age (aRR) with a 95% confidence interval (CI; see Tables S1-S4 in the Appendix). To control for potential confounding effects among factors, a multivariate analysis was then carried out including all factors with *p*-values <.05 from the univariate analysis and partial relative risks (pRR) were calculated.

## Results

Among our cohort of 927 women, frequencies of the prenatal outcomes distributed as follows: prenatal depression (*n* = 144), bipolar disorder (*n* = 47) and schizophrenia-related disorders (*n* = 7), or any-MI (*n* = 423), as well as prenatal depression comorbid with an anxiety disorder (*n* = 57), and bipolar disorder comorbid with an anxiety disorder (*n* = 19). The frequencies of the postpartum outcomes distributed as follows: postpartum depression (*n* = 239), bipolar disorder (*n* = 94), postpartum psychosis (*n* = 15), schizophrenia-related disorders (*n* = 10), any-MI (*n* = 521), and comorbid conditions such as postpartum depression with anxiety (*n* = 78), personality (*n* = 36), or obsessive-compulsive (*n* = 32) disorders, as well as bipolar disorder with anxiety (*n* = 24) or personality (*n* = 17) disorders.

Table 1 presents the psychiatric, sociodemographic, medical, and obstetric factors significantly associated with SMIs or any-MI diagnosed during pregnancy and postpartum according to univariate and multivariate analyses, ranked by effect size (aRR). Table 2 focuses on factors related to SMIs with comorbidity.

**Table 1.** Significant Factors Associated With New Cases of Depression, Bipolar Disorder, Schizophrenia-Related Disorder, or Any Disorder Diagnosed During Pregnancy or Postpartum Among the 927 and 772 Women Followed up During Pregnancy and Postpartum, Respectively. Effect Sizes are Shown in Terms of Relative Risks Adjusted for Age Both in Univariate (aRR) and Multivariate (pRR) Analyses. Associated Factors are Ranked According to Their aRR Effect Sizes

New dx during pregnancy	Associated factors	Relative risk (RR)		
		aRR	pRR^a^	95% CI
Prenatal depression (*n* = 144)				
	Premenstrual dysphoric disorder history	2.17**		
	No prior psychotropic use (before consultation)^b^	1.89***	2.42***	[1.46, 4.03]
	Musculoskeletal/rheumatological medical history^b,c^	1.48*	1.54*	[1.04, 2.29]
	Depressive disorder history	1.46*	1.56*	[1.05, 2.31]
	Age at childbirth >30	1.44*		
	No psychotropic use during pregnancy	1.44*		
Bipolar disorder (*n* = 47)				
	Past postpartum psychosis history^b,c^	8.13***	11.45***	[3.41, 38.46]
	Past intrusive thoughts of harming the infant	3.82*		
	Psychotic subclinical symptoms history	2.62*		
Schizophrenia-related disorder (*n* = 7)				
	Psychotic subclinical symptoms history^d^	43.96***		
	Psychiatric hospitalization history	15.15**		
	Cluster C personality disorder history	13.75*		
	Adjustment disorder history^d^	13.04**	7.19*	[1.16, 24.70]
	Unplanned and/or unwanted pregnancy	10.03*		
	Marital status (single)^b^	5.74*	6.68*	[1.04, 28.21]
Any-MI (*n* = 423)				
	Cluster C personality disorder history^b^	1.54*	1.30***	[1.15, 1.47]
	Premenstrual dysphoric disorder history	1.36*		
	Unplanned and/or unwanted pregnancy	1.35***		
	Cluster C personality traits	1.22**		
	Multiparous	1.17*	1.15*	[1.03, 1.30]
	No prior psychotropic use (before consultation)^b^	1.11*	1.13*	[1.01, 1.26]

New dx during postpartum	Associated factors	Relative risk (RR)		
		aRR	pRR	95% CI
Postpartum depression (*n* = 239)				
	No new diagnosis during pregnancy^e^	2.69***	2.11**	[1.32, 3.34]
	No psychotropic use during pregnancy	2.13***		
	No past psychiatric diagnosis^e^	1.63***	1.97*	[1.16, 3.37]
	No medical history	1.30*		
	Cluster C personality traits	1.24*		
	Age at childbirth ≤ 30	1.23*		
Bipolar disorder (*n* = 94)				
	No new diagnosis during pregnancy^e^	3.50***	3.38***	[1.94, 5.88]
	(Hypo)manic subclinical symptoms history^e,c^	3.39**	3.26***	[1.72, 6.15]
Postpartum psychosis (*n* = 15)				
	No past psychiatric diagnosis^e^	7.14**	N/A	[1.79, 25.00]
Schizophrenia-related disorder (*n* = 10)				
	Psychotic subclinical symptoms during pregnancy	41.51***		
	Psychotic subclinical symptoms history^d^	14.45***	9.38*	[1.15, 85.33]
	Unplanned and/or unwanted pregnancy	11.99*		
	Psychiatric hospitalization (history or pregnancy)	8.07***		
	Adjustment disorder history^d^	6.13*		
	Cluster B personality disorder history	5.33*		
Any-MI (*n* = 521)				
	Bipolar disorder history	2.26***	2.36**	[1.40, 3.98]
	No new diagnosis during pregnancy^e^	1.73***	2.30***	[1.79, 2.96]
	Somatic related disorder history	1.58***		
	No past psychiatric diagnosis	1.34***		
	Multiparous	1.16**		

dx, diagnosis; aRR, age adjusted relative risks (univariate analyses); pRR, partial relative risks obtained from the modified Poisson regression model adjusted for age and all other associated factors (multivariate analyses); N/A, not applicable due to only one significant factor or due to a rare factor involved.

*P ≤ .05 **P ≤ .01 ***P ≤ .001.

^a^When the number of significant factors identified in the univariate analyses, multiplied by ten, exceeded the number of subjects with a given outcome, only pairs of factors were included in the multivariate Poisson regression model. For each factor, the model with the lowest pRR among all models including that factor was retained.

^b^Factor specific to pregnancy regardless of SMI.

^c^Shared by SMI with/without comorbidity.

^d^Factor specific to SMI regardless of timing of diagnosis.

^e^Factor specific to postpartum regardless of SMI.

**Table 2.** Significant Factors Associated With Disorders Diagnosed During Pregnancy or Postpartum, Which are Comorbid^1^ With a SMI Among the 927 and 772 Women Followed Up During Pregnancy and Postpartum, Respectively. Effect Sizes are Shown in Terms of Relative Risks Adjusted for Age Both in Univariate (aRR) and Multivariate (pRR) Analyses. Factors are Ranked According to Their aRR Effect Sizes

New dx during pregnancy	Associated factors	Relative risk (RR)		
		aRR	pRR^a^	95% CI
Prenatal depression + anxiety disorder (*n* = 57)				
	Musculoskeletal/rheumatological medical history^b,c^	2.05*	1.77*	[1.01, 3.14]
	Foreign nativity (outside Canada)^d^	1.94*	2.41**	[1.27, 4.60]
	No psychotropic use during pregnancy^d^	1.92*	1.93*	[1.12, 3.34]
Bipolar disorder + anxiety disorder (*n* = 19)				
	Past postpartum psychosis history^b,c^	16.67**	N/A	[2.44, 100.00]

New dx during postpartum	Associated factors	Relative risk (RR)		
		aRR	pRR	95% CI
Postpartum depression + anxiety disorder (*n* = 78)				
	No new diagnosis during pregnancy^e^	10.26***	9.74**	[2.25, 42.11]
	Somatic related disorder history	3.97*		
	No psychotropic use during pregnancy^d^	2.41*	4.05**	[1.47, 11.16]
	Hypersensitivity to hormone fluctuations history	2.20**		
	Anxiety subclinical symptoms history^e^	2.03**	3.47*	[1.08, 11.15]
	Cluster C personality traits	1.86**	3.66**	[1.38, 9.72]
	Foreign nativity (outside Canada)^d^	1.75*	2.96*	[1.13, 7.77]
	Depressive disorder history	1.69*		
Postpartum depression + personality disorder (*n* = 36)				
	Past intrusive thoughts of harming the infant	12.91**	N/A	[1.89, 100.00]
	No new diagnosis during pregnancy	4.07**	N/A	[1.61, 10.00]
Postpartum depression + obsessive-compulsive disorder (*n* = 32)				
	No new diagnosis during pregnancy^e^	22.16**	18.56**	[2.72, 126.74]
	Somatic related disorder history^e^	9.02***	4.91*	[1.19, 20.22]
	Hypersensitivity to hormone fluctuations history^e^	4.19***	2.93*	[1.02, 8.40]
Bipolar disorder + anxiety disorder (*n* = 24)				
	(Hypo)manic subclinical symptoms history^e,c^	7.50***	8.11*	[1.32, 49.91]
	Past intrusive thoughts of harming the infant	4.14*		
	No new diagnosis during pregnancy	2.76*		
	Depressive disorder history	2.74*		
Bipolar disorder + personality disorder (*n* = 17)				
	(Hypo)manic subclinical symptoms history^e,c^	7.51**	5.97*	[1.17, 30.47]
	Past suicidal behavior^e^	4.16**	3.86*	[1.27, 11.69]

dx, diagnosis; aRR, age adjusted relative risks (univariate analyses); pRR, partial relative risks obtained from the modified Poisson regression model adjusted for age and all other associated factors (multivariate analyses); N/A, not applicable due to only one significant factor or due to a rare factor involved.

*P ≤ .05 **P ≤ .01 ***P ≤ .001.

^a^When the number of significant factors identified in the univariate analyses, multiplied by ten, exceeded the number of subjects with a given outcome, only pairs of factors were included in the multivariate Poisson regression model. For each factor, the model with the lowest pRR among all models including that factor was retained.

^b^Factor specific to pregnancy regardless of SMI.

^c^Shared by SMI with/without comorbidity.

^d^Factor specific to SMI regardless of timing of diagnosis.

^e^Factor specific to postpartum regardless of SMI.

## Discussion

Our study aimed to reveal factors associated with SMIs diagnosed either during pregnancy or within one year postpartum, with or without psychiatric comorbidity, in help-seeking women. We used multivariate analyses to reduce redundancy and highlight factors that remained significant after adjusting for their confounding effects with other factors. Only these resulting factors are interpreted in the following discussion, firstly paying attention to those that are specific to a perinatal period, followed by those that are specific to a given SMI.

Five factors were specifically associated with a new disorder during pregnancy, regardless of the diagnosis (b in Tables 1-2): being single, no prior psychotropic use, a history of musculoskeletal/rheumatological conditions, and cluster C personality disorder. Additionally, history of postpartum psychosis in multiparous women was strongly associated with bipolar disorder, supporting evidence that postpartum psychosis often evolves into bipolar disorder.^47-49^

Seven factors were specifically associated with new disorders during postpartum, regardless of the diagnosis (e in Tables 1-2): gonadal hormone hypersensitivity, history of anxiety or (hypo)manic subclinical symptoms or somatic related disorder, past suicidal behavior, no past psychiatric diagnosis, and no new psychiatric disorder during pregnancy. The latter two suggest that the postpartum period may trigger psychiatric disorders in women without prior vulnerability. Although previous studies found a past psychiatric history to be a factor associated with postpartum depression,^10,26,50^ our high prevalence of psychiatric history (73.3%) in our help-seeking women cohort may explain this turnaround, suggesting that proper psychiatric follow-up could act as a preventive factor.^51-53^ Our findings of factors specifically associated to each perinatal period support the idea that psychiatric disorders diagnosed during pregnancy or postpartum may have distinct etiologies and phenotypes.^11,54,55^ However, prospective studies with additional measures are needed to distinguish new-onset perinatal disorders from previously unrecognized or untreated pre-existing symptoms.

We then identified specific factors for a given SMI, regardless of the timing of diagnosis (d in Tables 1-2). Foreign nativity (outside Canada) and no psychotropic use during pregnancy were associated with a depression comorbid with anxiety, both during pregnancy and postpartum. The latter finding highlights the critical importance of effective perinatal treatment, and its associated medical monitoring, in preventing the adverse consequences of untreated maternal psychiatric illness.^56,57^ However, we were unable to fully assess the impact of psychotropic use, as the absence of pharmacotherapy did not necessarily indicate a lack of care (eg, psychotherapy data were unavailable). The clinic follows a conservative treatment approach, emphasizing monotherapy, the lowest effective dose, and the combination of pharmacological and non-pharmacological interventions. Moreover, a history of psychotic subclinical symptoms, and adjustment disorder seems to predict schizophrenia-related disorders in both periods. Our results thus suggest that some factors are specifically associated with a perinatal period (pregnancy or postpartum), regardless of the diagnosis, whereas others are associated with a given disorder, regardless of the timing of diagnosis. In contrast, other factors were non-specific to a SMI but rather associated with any-MI: a history of bipolar or cluster C personality disorders, multiparity, no prior psychotropic use before consultation, and no new psychiatric disorder during pregnancy.

Most previous studies have focused on depression, so our findings on factors associated with perinatal-diagnosed SMIs with comorbidity provide a broader perspective. In our cohort, we identified some factors associated with a specific SMI, both with and without comorbidity c in Tables 1-2), such as musculoskeletal/rheumatological conditions for prenatal depression, and past postpartum psychosis for bipolar disorder diagnosed during pregnancy. In contrast, the absence of a past psychiatric diagnosis was a factor only associated with SMIs without comorbidities, whereas a history of psychiatric vulnerability (history of anxiety or (hypo)manic subclinical symptoms or suicidal behavior or somatic related disorder or cluster C personality traits or gonadal hormone hypersensitivity) appeared to predict the development of more complex SMIs, ie, those with comorbidities. Moreover, foreign nativity was a sociodemographic factor that appeared to contribute to disease complexity, specifically in cases of a SMI with comorbidity.

Our data highlight that the perinatal period is a time of increased vulnerability to a range of mental health disorders, not just depression. This underscores the need for the development and validation of broader screening tools, as well as timely and appropriate care, and integrated mental health services equivalent to physical health follow-up, despite the current lack of clear recommendations from official bodies, calling for a shift in mindset and stronger collaboration among all stakeholders.

### Limitations and Strengths

Our cohort consisted only of women receiving perinatal psychiatric care for past or active psychiatric conditions, which may limit the generalizability to the broader female population. Consequently, our findings are specifically relevant to clinical populations and may not fully capture the spectrum of experiences in all women. Focusing on women with disorders, we did not compare them to those without psychiatric conditions, unlike general population studies, which may limit result interpretation. Also, retrospective chart reviews cannot definitively determine onset, and past psychiatric history can be underreported due to stigma, lack of awareness, and absence of formal diagnosis or treatment. The accuracy of previous personality disorder diagnoses could be limited, as such conditions are often underassessed, underdiagnosed, or undisclosed. Additionally, we did not have access to data on alcohol use, socioeconomic status, or a history of abuse that could have been associated factors. Despite these limitations, this study provides valuable insights from a cohort of 927 help-seeking women, focusing on factors associated with various SMIs diagnosed through a clinical follow-up, unlike previous studies that mostly screened for depression.^58^ Our findings on factors associated with bipolar and schizophrenia-related disorders diagnosed during pregnancy or postpartum are groundbreaking, as most prior research has focused on perinatal relapse in women with these preexisting conditions.

## Conclusion

In conclusion, this study offers valuable insights into perinatal mental health, particularly for women receiving specialized care for past or active psychiatric conditions. Our findings highlight specific factors associated with SMIs occurring during pregnancy or postpartum, with and without comorbidities, thus explaining why some trajectories were more likely than others among those revealed in Painchaud et al (2025).^7^ Few social vulnerability factors, such as being single or of foreign natality, were specifically associated to SMI diagnoses during the perinatal period, whereas a greater number of psychiatric vulnerability factors showed significant associations. Improving our understanding of factors associated with psychiatric conditions in the perinatal period is crucial for prevention, better care and outcomes for affected women.

## Supplemental Material

Suppplemental Material - Factors Associated With Severe Mental Illnesses Newly Diagnosed in Perinatal Psychiatric Care: Findings From a Large Clinical Cohort Suppplemental Material for Factors Associated With Severe Mental Illnesses Newly Diagnosed in Perinatal Psychiatric Care: Findings From a Large Clinical Cohort by Alexandra Painchaud, Marie-Josée Poulin, Célia Matte-Gagné and Chantal Mérette in Annals of Clinical Psychiatry
