# Supplementary material for: Factors Associated With Severe Mental Illnesses Newly Diagnosed in Perinatal Psychiatric Care: Findings From a Large Clinical Cohort
Source: Ann Clin Psychiatry. 2026 Mar 12;37(2):79–87. doi: 10.1177/10401237261434895 (PMC13050553; doi:10.1177/10401237261434895)
Supplement: Suppplemental Material - Factors Associated With Severe Mental Illnesses Newly Diagnosed in Perinatal Psychiatric Care: Findings From a Large Clinical Cohort [file sj-pdf-1-acy-10.1177_10401237261434895.pdf]

Appendix

Table S1

Factors associated with new[a] disorders diagnosed during pregnancy *among the cohort of women who required psychiatric care in a perinatal psychiatry clinic and were followed up to pregnancy (n = 927): prenatal depression (n = 144), bipolar disorder[b] (n = 47), SZ-related disorder[c] (n = 7) and any psychiatric disorder[d] (n = 423). According to Poisson regression models that included one factor at a time and were adjusted for the mother's age at childbirth, an adjusted relative risk (aRR) was estimated for each factor.*

Associated factors[e]			New incidence during **pregnancy**[f]											
			**Prenatal depression** n = 144			**Bipolar disorder** n = 47			**SZ-related** n = 7			**Any diagnosis** n = 423		
		**N**	**prop**	**$_{a}$RR[g]**	**95% CI**	**prop**	**$_{a}$RR**	**95% CI**	**prop**	**$_{a}$RR**	**95% CI**	**prop**	**$_{a}$RR**	**95% CI**
**<u>Past psychiatric history</u>**														
Depressive disorder (any one)	Yes	335	0.19	**1.46***	[1.04, 2.04]	0.06	1.23	[0.65, 2.32]	0.01	0.63	[0.10, 3.84]	0.51	1.14	[0.97, 1.32]
	No	413	0.13			0.05			0.01			0.43		
Prenatal depression[h]	Yes	11	0.36	1.89	[0.83, 4.17]	0.00	0.00	-	0.00	0.00	-	0.45	0.97	[0.50, 1.89]
	No	348	0.19			0.06			0.01			0.47		
Postpartum depression[h]	Yes	91	0.22	1.15	[0.72, 1.82]	0.04	0.69	[0.24, 1.96]	0.00	0.00	-	0.40	0.80	[0.61, 1.06]
	No	268	0.19			0.06			0.01			0.49		
Premenstrual dysphoric disorder	Yes	23	0.35	**2.17****	[1.22, 3.85]	0.04	0.83	[0.12, 5.88]	0.00	0.00	-	0.65	**1.36***	[1.01, 1.85]
	No	865	0.15			0.05			0.01			0.47		
Hypersensitivity to gonadal hormone fluctuations	Yes	55	0.15	0.90	[0.47, 1.72]	0.05	1.05	[0.34, 3.33]	0.00	0.00	-	0.58	1.25	[0.98, 1.59]
	No	833	0.16			0.05			0.01			0.46		
Depressive subclinical symptoms	Yes	152	0.17	1.08	[0.63, 1.37]	0.06	1.18	[0.42, 1.72]	0.01	0.81	[0.15, 10.16]	0.53	1.14	[0.96, 1.35]
	No	736	0.16			0.05			0.01			0.46		
Bipolar disorder	Yes	85	N/A	N/A	N/A	N/A	N/A	N/A	0.01	1.59	[0.18, 14.42]	0.32	0.64	[0.46, 0.88]
	No	663	N/A			N/A			0.01			0.49		
(Hypo)/manic subclinical symptoms	Yes	14	0.00	0.00	-	0.07	1.39	[0.11, 5.00]	0.00	0.00	-	0.29	0.60	[0.25, 1.39]
	No	874	0.16			0.05			0.01			0.47		
Psychotic disorder														
Postpartum psychosis[h]	Yes	5	0.00	0.00	-	0.40	**8.13*****	[2.44, 25.00]	0.00	0.00	-	0.40	0.85	[0.29, 2.50]
	No	302	0.20			0.05			0.003			0.47		
SZ-related disorder	Yes	28	0.14	0.99	[0.39, 2.51]	0.07	1.45	[0.37, 5.61]	N/A	N/A	N/A	0.39	0.88	[0.54, 1.43]
	No	720	0.16			0.05			N/A			0.47		
Psychotic subclinical symptoms	Yes	48	0.02	0.12	[0.02, 0.85]	0.13	**2.62***	[1.18, 5.88]	0.10	**43.96*****	[9.09, 250.0]	0.38	0.78	[0.54, 1.14]
	No	840	0.17			0.05			0.002			0.48		
Anxiety disorder (any one)	Yes	204	0.14	0.87	[0.59, 1.29]	0.05	0.95	[0.47, 1.92]	0.005	0.54	[0.07, 4.42]	0.49	1.04	[0.88, 1.23]
	No	544	0.16			0.05			0.01			0.46		
Anxiety subclinical symptoms	Yes	154	0.14	0.85	[0.77, 1.77]	0.05	1.00	[0.47, 2.12]	0.00	0.00	-	0.51	1.10	[0.93, 1.30]
	No	734	0.16			0.05			0.01			0.46		
Obsessive compulsive disorder	Yes	54	0.15	0.96	[0.50, 1.86]	0.04	0.72	[0.18, 2.89]	0.00	0.00	-	0.43	0.92	[0.67, 1.25]
	No	694	0.16			0.05			0.01			0.47		

Intrusive thoughts of harming the infant[h]	Yes	15	0.13	0.66	[0.18, 2.44]	0.20	**3.82***	[1.27,11.11]	0.00	0.00	-	0.33	0.70	[0.34, 1.45]
	No	344	0.20			0.05			0.01			0.47		
Post-traumatic stress disorder	Yes	45	0.07	0.43	[0.14, 1.32]	0.04	0.87	[0.22, 3.50]	0.02	3.05	[0.30, 31.45]	0.49	1.09	[0.79, 1.49]
	No	703	0.16			0.05			0.01			0.47		
Adjustment disorder	Yes	54	0.15	0.93	[0.48, 1.80]	0.06	1.10	[0.35, 3.46]	0.06	**13.04****	[2.65, 64.29]	0.56	1.19	[0.93, 1.54]
	No	694	0.16			0.05			0.004			0.46		
Personality disorder	Yes	72	0.10	0.61	[0.30, 1.27]	0.04	0.81	[0.26, 2.53]	0.03	4.64	[0.89, 24.13]	0.44	0.96	[0.74, 1.27]
(any one)	No	676	0.16			0.05			0.01			0.47		
Cluster B	Yes	64	0.09	0.60	[0.27, 1.30]	0.05	0.92	[0.35, 3.40]	0.02	2.08	[0.06, 4.01]	0.41	0.88	[0.65, 1.19]
	No	684	0.16			0.05			0.01			0.47		
Cluster C	Yes	11	0.09	0.56	[0.27, 11.40]	0.00	0.00	-	0.09	**13.75***	[1.67, 100.0]	0.73	**1.54***	[1.06, 2.22]
	No	737	0.16			0.05			0.01			0.46		
Personality traits	Yes	538	0.17	1.06	[0.78, 1.44]	0.06	1.28	[0.71, 2.30]	0.01	0.50	[0.12, 2.19]	0.51	**1.21***	[1.04, 1.40]
(any one)	No	356	0.15			0.04			0.01			0.42		
Cluster B	Yes	248	0.13	0.75	[0.52, 1.08]	0.07	1.61	[0.92, 2.86]	0.004	0.43	[0.05, 3.57]	0.49	1.05	[0.90, 1.22]
	No	646	0.17			0.04			0.01			0.47		
Cluster C	Yes	411	0.18	1.15	[0.85, 1.56]	0.06	1.12	[0.64, 1.96]	0.005	0.48	[0.10, 2.38]	0.53	**1.22****	[1.06, 1.41]
	No	483	0.15			0.05			0.01			0.43		
Attention-deficit/ hyperactivity disorder	Yes	77	0.09	0.67	[0.28, 1.16]	0.06	1.30	[0.53, 3.23]	0.00	0.00	-	0.45	0.99	[0.78, 1.27]
	No	811	0.17			0.05			0.01			0.47		
Somatic related disorder	Yes	9	0.11	0.67	[0.11, 4.21]	0.00	0.00	-	0.00	0.00	-	0.44	0.91	[0.43, 1.92]
	No	739	0.16			0.05			0.01			0.47		
Eating disorder	Yes	75	0.16	1.10	[0.64, 1.91]	0.07	1.38	[0.55, 3.44]	0.01	1.74	[0.23, 13.42]	0.45	0.99	[0.76, 1.28]
	No	673	0.16			0.05			0.01			0.47		
Sleeping disorder	Yes	7	0.00	0.00	-	0.00	0.00	-	0.00	0.00	-	0.57	1.25	[0.66, 2.38]
	No	741	0.16			0.05			0.01			0.47		
Addictive disorder	Yes	150	0.13	0.83	[0.52, 1.32]	0.05	0.91	[0.40, 2.05]	0.02	3.97	[0.94, 16.76]	0.48	1.09	[0.90, 1.32]
	No	598	0.16			0.05			0.01			0.46		
**Suicidal ideation**	Yes	194	0.15	0.97	[0.67, 1.41]	0.06	1.27	[0.67, 2.38]	0.01	1.41	[0.28, 7.14]	0.50	1.09	[0.93, 1.28|
	No	694	0.16			0.05			0.01			0.46		
**Suicidal behavior**	Yes	95	0.13	0.81	[0.47, 1.39]	0.03	0.58	[0.18, 1.89]	0.01	1.30	[0.17, 10.00]	0.54	1.20	[0.98, 1.47]
	No	793	0.16			0.05			0.01			0.46		
**No psychiatric history**	Yes	91	0.18	1.18	[0.73, 1.89]	0.02	0.40	[0.10, 1.64]	0.00	0.00	-	0.41	0.87	[0.67, 1.12]
	No	657	0.15			0.05			0.01			0.48		
**Any psychiatric history in a previous perinatal period**[h]	Yes	164	0.21	1.11	[0.73, 1.67]	0.07	1.45	[0.64, 3.23]	0.01	1.23	[0.07, 20.00]	0.42	0.84	[0.67, 1.05]
	No	198	0.19			0.05			0.01			0.50		
**Psychiatric hospitalization**														
Before consultation (history)	Yes	128	0.08	0.44	[0.24, 0.88]	0.07	1.41	[0.70, 2.86]	0.04	**15.15****	[2.94, 100.0]	0.39	0.80	[0.63, 1.01]
	No	766	0.17			0.05			0.003			0.49		
**No psychotropic medication**														
Before consultation	Yes	228	0.36	**1.89*****	[1.37, 2.63]	0.07	0.68	[0.37, 1.22]	0.004	0.23	[0.03, 2.27]	0.87	**1.11***	[1.02, 1.20]
	No	219	0.19			0.11			0.02			0.79		
During pregnancy	Yes	208	0.28	**1.44***	[1.05, 1.96]	0.06	0.69	[0.36, 1.32]	0.005	0.40	[0.04, 3.85]	0.62	0.94	[0.83, 1.08]
	No	345	0.19			0.08			0.01			0.66		

**Family psychiatric history**														
	Yes	676	0.16	0.71	[0.45, 1.11]	0.06	2.12	[0.52, 8.58]	0.01	-	-	0.48	1.01	[0.79, 1.29]
	No	75	0.23			0.03			0.00			0.48		
**Sociodemographic**														
Age at childbirth[i]	>30	455	0.19	**1.44***	[1.05, 1.94]	0.05	1.10	[0.63, 1.92]	0.004	0.39	[0.08, 1.98]	0.52	1.13	[0.89, 1.42]
	≤30	439	0.13			0.05						0.42		
Nativity	Yes	810	0.15	0.67	[0.44, 1.02]	0.05	0.88	[0.36, 2.19]	0.01	-	-	0.48	1.15	[0.89, 1.49]
(Born in Canada)	No	84	0.24			0.06			0.00			0.43		
Marital status	Yes	99	0.13	0.83	[0.49, 1.41]	0.03	0.54	[0.17, 1.69]	0.03	**5.74***	[1.32, 25.00]	0.43	0.93	[0.74, 1.18]
(Single)	No	779	0.17			0.06			0.01			0.48		
**Medical history**														
Cardiopulmonary	Yes	100	0.09	0.53	[0.28, 1.02]	0.08	0.63	[0.30, 1.33]	0.01	2.04	[0.06, 4.12]	0.45	0.91	[0.72, 1.15]
	No	618	0.16			0.05			0.005			0.49		
Gastrointestinal	Yes	48	0.10	0.65	[0.28, 1.54]	0.04	0.75	[0.19, 3.03]	0.02	4.55	[0.54, 33.33]	0.42	0.85	[0.61, 1.19]
	No	670	0.16			0.06			0.004			0.49		
Neurological	Yes	156	0.14	0.88	[0.73, 1.75]	0.04	0.79	[0.57, 2.82]	0.01	1.19	[0.08, 8.66]	0.49	1.02	[0.86, 1.22]
	No	562	0.16			0.06			0.01			0.48		
Musculoskeletal/ rheumatological	Yes	142	0.21	**1.48***	[1.01, 2.17]	0.06	1.04	[0.45, 2.05]	0.00	0.00	-	0.49	1.02	[0.84, 1.22]
	No	576	0.14			0.05			0.01			0.48		
Endocrinal	Yes	78	0.18	1.15	[0.52, 1.45]	0.05	0.93	[0.39, 2.91]	0.01	2.70	[0.04, 3.41]	0.54	1.11	[0.89, 1.39]
	No	640	0.15			0.05			0.005			0.47		
Other physical illness	Yes	374	0.16	1.01	[0.71, 1.42]	0.06	1.32	[0.71, 2.48]	0.01	2.75	[0.26, 29.53]	0.51	1.13	[0.97, 1.31]
	No	344	0.15			0.05			0.003			0.45		
**Any medical history**	Yes	541	0.16	1.06	[0.62, 1.41]	0.05	0.95	[0.53, 2.13]	0.01	-	-	0.49	1.04	[0.87, 1.25]
	No	177	0.14			0.06			0.00			0.46		
**Obstetric (current pregnancy)**														
Multiparous	Yes	362	0.20	1.33	[0.98, 1.82]	0.06	1.22	[0.69, 2.17]	0.006	0.60	[0.11, 3.23]	0.54	**1.17***	[1.02, 1.35]
	No	507	0.14			0.05			0.01			0.44		
Unplanned/ unwanted pregnancy	Yes	217	0.19	1.25	[0.87, 1.79]	0.06	1.01	[0.49, 1.98]	0.02	**10.03***	[1.33, 100.0]	0.59	**1.35*****	[1.16, 1.56]
	No	422	0.16			0.05			0.002			0.45		
Complications during pregnancy	Yes	248	0.19	1.17	[0.69, 1.97]	0.08	1.49	[0.57, 3.87]	0.02	1.66	[0.19, 14.24]	0.58	1.04	[0.85, 1.28]
	No	99	0.16			0.05			0.01			0.56		
**Obstetric (previous pregnancies, if applicable)**														
Abortion	Yes	182	0.13	0.70	[0.47, 1.06]	0.04	0.64	[0.29, 1.39]	0.01	0.67	[0.08, 5.88]	0.47	0.95	[0.81, 1.14]
	No	595	0.18			0.06			0.01			0.49		
Miscarriage	Yes	197	0.15	0.83	[0.57, 1.20]	0.07	1.33	[0.69, 2.56]	0.01	0.61	[0.08, 4.55]	0.47	0.94	[0.79, 1.12]
	No	579	0.18			0.05			0.01			0.49		
Complications during pregnancy[h]	Yes	168	0.20	0.99	[0.53, 1.86]	0.05	0.90	[0.25, 3.23]	0.00	-	-	0.46	1.16	[0.80, 1.69]
	No	50	0.20			0.06			0.00			0.40		
Complications during childbirth[h]	Yes	99	0.14	0.58	[0.30, 1.12]	0.08	1.13	[0.37, 3.43]	0.00	-	-	0.41	0.91	[0.63, 1.31]
	No	57	0.25			0.07			0.00			0.46		

prop: proportion of women with a given outcome; $_{a}$**RR**: Adjusted relative risks, i.e. ratio of the proportion of women with a given outcome among those exposed over that proportion among those unexposed to the factor; **CI**: Confidence intervals.

[a]The psychiatric disorders newly diagnosed during pregnancy refer to disorders that were not reported in the woman's past history, or a new psychiatric episode when a previous one was completely resolved before pregnancy.

[b]Bipolar disorder included: type I, type II, atypical or unspecified, or cyclothymic disorder.
[c]SZ-related disorder included: schizophrenia, schizoaffective disorders, and substance-induced psychotic disorder.
[d]Any psychiatric disorder included the diagnosis of at least one psychiatric disorder (any one) diagnosed during pregnancy.
[e] Due to missing data in medical records, the sum of the number of women having (or not) a given risk factor does not always add up to 927.
[f]A woman can contribute to the prevalence of more than one diagnosis. Hence, each diagnosis is treated as a dichotomous variable (presence vs absence) and consequently, the sum of the percentages is not interpretable and can exceed 100%.
[g] aRR is the adjusted Relative Risk, i.e., the risk of the incidence of a new specific disorder during pregnancy among women having a given risk factor over that risk among women not having the risk factor, adjusted for the age of the mother at childbirth, based on a Poisson Regression model.
[h] These factors only apply to the multiparous women who have already had another child before this follow-up at the perinatal psychiatry clinic.
[i]This factor has not been adjusted for maternal age at childbirth.

* $p \leq 0.05$ ** $p \leq 0.01$ ***$p \leq 0.001$

Table S2

Factors associated with new[a] disorders diagnosed during postpartum *among the cohort of women who required psychiatric care in a perinatal psychiatry clinic and were followed up to postpartum (n = 772): postpartum depression (n = 239), bipolar disorder[b] (n = 94), postpartum psychosis (n = 15), schizophrenia-related disorder[c] (n = 10) and any psychiatric disorder[d] (n = 521). According to Poisson regression models that included one factor at a time and were adjusted for the mother's age at childbirth, an adjusted relative risk (aRR) was estimated for each factor.*

Associated factors[e]		New incidence during **postpartum**[f]															
			**Postpartum depression** n = 239			**Bipolar disorder** n = 94			**Postpartum psychosis** n = 15			**SZ-related** n = 10			**Any diagnosis** n = 521		
		**N**	**prop**	$_{a}$**RR**[g]	**95% CI**	**prop**	$_{a}$**RR**	**95% CI**	**prop**	$_{a}$**RR**	**95% CI**	**prop**	$_{a}$**RR**	**95% CI**	**prop**	$_{a}$**RR**	**95% CI**
**Past psychiatric history**																	
Depressive disorder (any one)	Yes	270	0.29	0.98	[0.77, 1.26]	0.11	1.07	[0.69, 1.66]	0.004	0.19	[0.02, 1.47]	0.01	0.64	[0.13, 3.04]	0.66	1.00	[0.90, 1.12]
	No	339	0.30			0.11			0.02			0.01			0.68		
Prenatal depression[h]	Yes	8	0.25	0.74	[0.21, 2.63]	0.13	1.10	[0.17, 7.14]	0.00	0.00	-	0.00	0.00	-	0.88	1.20	[0.90, 1.61]
	No	292	0.34			0.11			0.01			0.01			0.72		
Postpartum depression[h]	Yes	72	0.36	1.16	[0.81, 1.67]	0.07	0.55	[0.22, 1.37]	0.00	0.00	-	0.00	0.00	-	0.79	1.14	[0.98, 1.32]
	No	228	0.32			0.13			0.01			0.10			0.71		
Premenstrual dysphoric disorder	Yes	17	0.18	0.56	[0.20, 1.56]	0.06	0.49	[0.07, 3.33]	0.00	0.00	-	0.00	0.00	-	0.47	0.68	[0.41, 1.14]
	No	716	0.32			0.12			0.02			0.01			0.69		
Hypersensitivity to hormone fluctuations	Yes	47	0.38	1.23	[0.85, 1.81]	0.06	0.52	[0.17, 1.59]	0.00	0.00	-	0.00	0.00	-	0.77	1.12	[0.95, 1.33]
	No	686	0.31			0.12			0.02			0.01			0.68		
Depressive subclinical symptoms	Yes	131	0.34	1.08	[0.72, 1.22]	0.07	0.52	[0.26, 1.01]	0.00	0.00	-	0.00	0.00	-	0.67	0.97	[0.85, 1.11]
	No	602	0.31			0.13			0.02			0.01			0.69		
Bipolar disorder	Yes	73	N/A	N/A	N/A	N/A	N/A	N/A	0.01	1.09	[0.14, 8.72]	0.00	0.00	-	0.32	**2.26*****	[1.61, 3.23]
	No	536	N/A			N/A			0.01			0.01			0.72		
(Hypo)/ manic subclinical symptoms	Yes	13	0.08	0.24	[0.04, 1.59]	0.38	**3.39****	[1.61, 7.14]	0.08	5.26	[0.67, 33.33]	0.00	0.00	-	0.54	0.79	[0.48, 1.30]
	No	720	0.32			0.12			0.02			0.01			0.69		
Psychotic disorder																	
Postpartum Psychosis[h]	Yes	5	0.00	0.00	-	0.00	0.00	-	0.00	0.00	-	0.00	0.00	-	0.20	0.28	[0.05, 1.64]
	No	249	0.33			0.10			0.01			0.01			0.73		
SZ-related disorder	Yes	23	0.13	0.41	[0.14, 1.15]	0.00	0.00	-	0.04	3.31	[0.47, 23.17]	N/A	N/A	N/A	0.30	0.43	[0.23, 0.79]
	No	586	0.30			0.12			0.01			N/A			0.68		
Psychotic subclinical symptoms	Yes	39	0.05	0.16	[0.04, 0.61]	0.13	1.09	[0.47, 2.50]	0.00	0.00	-	0.10	**14.45*****	[4.00,50.00]	0.51	0.74	[0.55, 1.00]
	No	694	0.33			0.12			0.02			0.01			0.70		
Anxiety disorder (any one)	Yes	172	0.17	0.51	[0.36, 0.72]	0.10	0.83	[0.49, 1.40]	0.02	1.53	[0.37, 6.31]	0.00	0.00	-	0.61	0.88	[0.77, 1.01]
	No	437	0.34			0.12			0.01			0.01			0.69		
Anxiety subclinical symptoms	Yes	128	0.32	1.04	[0.73, 1.27]	0.09	0.69	[0.38, 1.27]	0.00	0.00	-	0.01	0.61	[0.08, 4.55]	0.74	1.11	[0.99, 1.25]
	No	605	0.31			0.13			0.02			0.01			0.67		
Obsessive compulsive disorder	Yes	47	0.23	0.78	[0.46, 1.33]	0.15	1.36	[0.66, 2.80]	0.00	0.00	-	0.00	0.00	-	0.57	0.85	[0.66, 1.10]
	No	562	0.30			0.11			0.01			0.01			0.68		
Intrusive thoughts of harming the infant[h]	Yes	13	0.23	0.69	[0.26, 1.89]	0.15	1.39	[0.37, 5.26]	0.00	0.00	-	0.00	0.00	-	0.77	1.06	[0.79, 1.45]
	No	287	0.34			0.11			0.01			0.01			0.72		
Post-traumatic stress disorder	Yes	33	0.24	0.78	[0.43, 1.43]	0.09	0.77	[0.25, 2.30]	0.00	0.00	-	0.00	0.00	-	0.58	0.84	[0.62, 1.12]
	No	576	0.30			0.11			0.01			0.01			0.68		
Adjustment disorder	Yes	46	0.26	0.88	[0.54, 1.43]	0.02	0.18	[0.03, 1.28]	0.00	0.00	-	0.04	**6.13***	[1.15, 32.54]	0.67	1.01	[0.82, 1.24]
	No	563	0.30			0.12			0.01			0.01			0.67		
Personality disorder (any one)	Yes	56	0.16	0.50	[0.28, 0.91]	0.13	1.08	[0.52, 2.26]	0.00	0.00	-	0.04	4.91	[0.96, 25.22]	0.50	0.72	[0.55, 0.94]
	No	553	0.31			0.11			0.01			0.01			0.69		
Cluster B	Yes	52	0.17	0.54	[0.30, 0.98]	0.13	1.18	[0.56, 2.44]	0.00	0.00	-	0.04	**5.33***	[1.04, 25.00]	0.50	0.71	[0.54, 0.94]
	No	557	0.31			0.11			0.01			0.01			0.69		
Cluster C	Yes	8	0.00	0.00	-	0.00	0.00	-	0.00	0.00	-	0.00	0.00	-	0.50	0.76	[0.37, 1.56]
	No	601	0.30			0.11			0.01			0.01			0.67		

Personality traits	Yes	459	0.32	1.01	[0.82, 1.25]	0.12	0.95	[0.65, 1.40]	0.02	0.74	[0.27, 2.01]	0.01	0.28	[0.07, 1.06]	0.68	0.97	[0.88, 1.08]
(any one)	No	294	0.32			0.13			0.02			0.02			0.70		
Cluster B	Yes	210	0.23	0.64	[0.49, 0.84]	0.14	1.14	[0.76, 1.72]	0.01	0.63	[0.18, 2.22]	0.005	0.28	[0.04, 2.17]	0.69	0.99	[0.89, 1.10]
	No	543	0.35			0.12			0.02			0.02			0.69		
Cluster C	Yes	348	0.35	**1.24***	[1.00, 1.52]	0.12	0.92	[0.63, 1.35]	0.01	0.43	[0.14, 1.37]	0.01	0.30	[0.06, 1.41]	0.68	0.99	[0.90, 1.09]
	No	405	0.29			0.13			0.03			0.02			0.70		
Attention-deficit/	Yes	58	0.26	0.79	[0.51, 1.23]	0.10	0.83	[0.38, 1.82]	0.00	0.00	-	0.02	1.43	[0.17, 12.50]	0.62	0.89	[0.72, 1.09]
hyperactivity disorder	No	675	0.32			0.12			0.01			0.01			0.69		
Somatic related	Yes	6	0.50	1.89	[0.88, 4.06]	0.00	0.00	-	0.00	0.00	-	0.00	0.00	-	1.00	**1.58*****	[1.45, 1.71]
disorder	No	603	0.29			0.11			0.01			0.01			0.67		
Eating disorder	Yes	63	0.27	0.85	[0.56, 1.30]	0.19	1.74	[0.99, 3.05]	0.00	0.00	-	0.00	0.00	-	0.65	0.94	[0.78, 1.13]
	No	546	0.30			0.10			0.01			0.01			0.67		
Sleeping disorder	Yes	6	0.17	0.54	[0.09, 3.26]	0.17	1.43	[0.23, 9.09]	0.00	0.00	-	0.00	0.00	-	0.50	0.73	[0.33, 1.63]
	No	603	0.30			0.11			0.01			0.01			0.67		
Addictive disorder	Yes	112	0.19	0.55	[0.36, 0.82]	0.12	0.98	[0.55, 1.74]	0.00	0.00	-	0.01	0.86	[0.10, 7.45]	0.63	0.89	[0.76, 1.04]
	No	497	0.32			0.11			0.02			0.01			0.68		
**Suicidal ideation**	Yes	164	0.30	0.92	[0.71, 1.19]	0.14	1.20	[0.78, 1.85]	0.01	0.30	[0.04, 2.38]	0.01	0.97	[0.19, 5.00]	0.70	1.01	[0.90, 1.14]
	No	569	0.32			0.11			0.02			0.01			0.68		
**Suicidal behavior**	Yes	72	0.21	0.60	[0.38, 0.95]	0.14	1.10	[0.58, 2.04]	0.00	0.00	-	0.00	0.00	-	0.68	0.96	[0.81, 1.14]
	No	661	0.33			0.12			0.02			0.01			0.69		
**No psychiatric**	Yes	75	0.45	**1.63*****	[1.23, 2.17]	0.16	1.47	[0.84, 2.63]	0.05	**7.14****	[1.79, 25.00]	0.01	1.41	[0.17, 11.11]	0.87	**1.34*****	[1.20, 1.49]
**history**	No	534	0.27			0.11			0.01			0.01			0.64		
**Any psychiatric history in a previous**	Yes	132	0.37	1.16	[0.85, 1.59]	0.09	0.70	[0.36, 1.35]	0.00	0.00	-	0.01	0.70	[0.06, 7.69]	0.77	1.10	[0.96, 1.25]
**perinatal period[h]**	No	177	0.32			0.13			0.02			0.01			0.71		

Pregnancy-onset psychiatric disorders

Depressive disorder	Yes	175	0.19	0.56	[0.41, 0.77]	0.05	0.38	[0.19, 0.75]	0.01	0.80	[0.17, 3.73]	0.01	0.41	[0.06, 2.98]	0.53	0.72	[0.62, 0.84]
	No	551	0.36			0.14			0.02			0.01			0.74		
Prenatal depression	Yes	110	0.18	0.54	[0.36, 0.82]	0.04	0.28	[0.11, 0.75]	0.01	0.62	[0.08, 5.08]	0.01	0.73	[0.10, 5.36]	0.49	0.68	[0.56, 0.84]
	No	616	0.34			0.13			0.02			0.01			0.72		
Premenstrual	Yes	15	0.20	1.28	[0.45, 3.70]	0.00	0.00	-	0.00	0.00	-	0.00	0.00	-	0.53	1.19	[0.72, 1.92]
dysphoric disorder	No	337	0.15			0.05			0.01			0.01			0.45		
Hypersensitivity to	Yes	26	0.04	0.24	[0.03, 1.61]	0.08	1.56	[0.38, 6.67]	0.00	0.00	-	0.00	0.00	-	0.31	0.66	[0.37, 1.19]
hormone fluctuations	No	326	0.16			0.05			0.01			0.02			0.47		
Bipolar disorder	Yes	38	N/A	N/A	N/A	N/A	N/A	N/A	0.00	0.00	-	0.03	2.29	[0.29, 17.84]	0.42	0.60	[0.41, 0.88]
	No	688	N/A			N/A			0.02			0.01			0.70		
Psychotic disorder																	
SZ-related disorder	Yes	5	0.00	0.00	-	0.20	1.76	[0.32, 9.68]	0.00	0.00	-	N/A	N/A	N/A	0.80	1.18	[0.80, 1.75]
	No	721	0.32			0.12			0.02			N/A			0.69		
Psychotic subclinical	Yes	6	0.00	0.00	-	0.17	3.33	[0.53, 20.0]	0.00	0.00	-	0.33	**41.51*****	[7.69, 250.00]	0.33	0.73	[0.23, 2.27]
symptoms	No	346	0.16			0.05			0.01			0.01			0.46		
Anxiety disorder	Yes	149	0.15	0.41	[0.28, 0.62]	0.03	0.19	[0.07, 0.52]	0.00	0.00	-	0.01	0.50	[0.06, 3.99]	0.47	0.64	[0.53, 0.76]
(any one)	No	577	0.36			0.14			0.02			0.01			0.75		
Obsessive compulsive	Yes	46	0.17	0.55	[0.29, 1.04]	0.04	0.37	[0.09, 1.45]	0.00	0.00	-	0.00	0.00	-	0.39	0.56	[0.39, 0.81]
disorder	No	680	0.33			0.12			0.02			0.01			0.71		
Intrusive thoughts of	Yes	18	0.22	1.45	[0.59, 3.57]	0.00	0.00	-	0.00	0.00	-	0.00	0.00	-	0.33	0.72	[0.37, 1.41]
harming the infant	No	334	0.15			0.05			0.01			0.01			0.46		
Post-traumatic stress	Yes	20	0.15	0.48	[0.17, 1.37]	0.00	0.00	-	0.00	0.00	-	0.00	0.00	-	0.45	0.66	[0.41, 1.08]
disorder	No	706	0.32			0.12			0.02			0.01			0.70		
Adjustment disorder	Yes	21	0.29	0.90	[0.45, 1.77]	0.00	0.00	-	0.00	0.00	-	0.00	0.00	-	0.57	0.83	[0.57, 1.19]
	No	705	0.32			0.12			0.02			0.01			0.69		
Personality disorder	Yes	40	0.10	0.30	[0.12, 0.77]	0.05	0.41	[0.10, 1.61]	0.00	0.00	-	0.00	0.00	-	0.45	0.64	[0.45, 0.91]
(any one)	No	686	0.33			0.12			0.02			0.01			0.70		
Cluster B	Yes	10	0.20	0.61	[0.17, 2.13]	0.00	0.00	-	0.00	0.00	-	0.00	0.00	-	0.50	0.71	[0.38, 1.35]
	No	716	0.32			0.12			0.02			0.01			0.69		
Attention-deficit/	Yes	32	0.03	0.19	[0.03, 1.33]	0.03	0.58	[0.08, 4.35]	0.00	0.00	-	0.00	0.00	-	0.41	0.88	[0.57, 1.37]
hyperactivity disorder	No	320	0.17			0.05			0.01			0.02			0.46		

Somatic related disorder	Yes	8	0.00	0.00	-	0.00	0.00	-	0.00	0.00	-	0.00	0.00	-	0.13	0.18	[0.03, 1.15]
	No	718	0.32			0.12			0.02			0.01			0.69		
Eating disorder	Yes	5	0.20	0.69	[0.12, 4.11]	0.00	0.00	-	0.00	0.00	-	0.00	0.00	-	0.60	0.93	[0.46, 1.85]
	No	721	0.32			0.12			0.02			0.01			0.69		
**Suicidal ideation**	Yes	33	0.15	1.00	[0.43, 2.33]	0.09	1.92	[0.59, 6.25]	0.00	0.00	-	0.00	0.00	-	0.55	1.22	[0.88, 1.72]
	No	319	0.15			0.05			0.01			0.02			0.45		
**No new pregnancy onset psychiatric disorder**	Yes	427	0.43	**2.69*** **	[2.00, 3.57]	0.17	**3.50*** **	[2.00, 6.25]	0.02	2.78	[0.60, 12.50]	0.01	0.33	[0.08, 1.28]	0.84	**1.73*** **	[1.52, 1.96]
	No	299	0.16			0.05			0.01			0.02			0.48		
**Psychiatric hospitalization**																	
Before consultation (history) or during pregnancy	Yes	121	0.13	0.38	[0.24, 0.61]	0.11	0.85	[0.49, 1.47]	0.02	0.82	[0.18, 3.70]	0.05	**8.07** **	[2.33, 25.00]	0.50	0.70	[0.58, 0.84]
	No	632	0.35			0.13			0.02			0.01			0.73		
**No psychotropic medication**																	
Before consultation	Yes	98	0.47	0.81	[0.64, 1.03]	0.13	0.61	[0.34, 1.08]	0.01	0.30	[0.04, 2.27]	0.00	0.00	-	0.96	0.98	[0.94, 1.03]
	No	204	0.58			0.22			0.03			0.01			0.98		
During pregnancy	Yes	163	0.32	**2.13*** **	[1.47, 3.03]	0.06	0.62	[0.30, 1.32]	0.01	0.79	[0.07, 9.09]	0.00	0.00	-	0.61	1.15	[0.97, 1.37]
	No	261	0.15			0.09			0.01			0.02			0.52		
During postpartum	Yes	70	0.19	0.54	[0.33, 0.88]	0.03	0.20	[0.05, 0.81]	0.00	0.00	-	0.00	0.00	-	0.54	0.78	[0.62, 0.97]
	No	575	0.35			0.14			0.02			0.01			0.70		
**Family psychiatric history**																	
	Yes	552	0.31	0.75	[0.54, 1.04]	0.12	1.90	[0.72, 5.03]	0.01	0.80	[0.10, 6.16]	0.01	0.36	[0.03, 3.74]	0.67	0.85	[0.74, 0.99]
	No	65	0.40			0.06			0.02			0.02			0.77		
**Sociodemographic**																	
Age at childbirth[i]	≤30	387	0.35	**1.23***	[1.01, 1.54]	0.13	1.15	[0.79, 1.69]	0.02	0.93	[0.34, 2.50]	0.02	2.44	[0.64, 9.09]	0.73	1.01	[0.86, 1.18]
	>30	366	0.28			0.12			0.02			0.01			0.65		
Nativity (Born in Canada)	Yes	674	0.31	0.82	[0.60, 1.11]	0.13	2.01	[0.84, 4.79]	0.02	0.44	[0.13, 1.53]	0.01	0.99	[0.12, 8.09]	0.68	0.88	[0.77, 1.01]
	No	79	0.37			0.06			0.04			0.01			0.76		
Marital status (Single)	Yes	88	0.24	0.71	[0.48, 1.05]	0.15	1.16	[0.67, 2.00]	0.02	1.08	[0.25, 4.76]	0.03	3.03	[0.81, 11.11]	0.69	1.01	[0.87, 1.17]
	No	651	0.33			0.12			0.02			0.01			0.69		
**Medical history**																	
Cardiopulmonary	Yes	79	0.25	0.79	[0.53, 1.18]	0.06	0.54	[0.23, 1.32]	0.01	1.05	[0.13, 8.33]	0.00	0.00	-	0.58	0.83	[0.68, 1.01]
	No	510	0.32			0.12			0.01			0.01			0.70		
Gastrointestinal	Yes	40	0.35	1.15	[0.74, 1.79]	0.08	0.68	[0.22, 2.04]	0.03	2.22	[0.28, 16.67]	0.00	0.00	-	0.78	1.15	[0.96, 1.37]
	No	549	0.31			0.11			0.01			0.01			0.68		
Neurological	Yes	127	0.21	0.63	[0.44, 0.90]	0.10	0.92	[0.52, 1.64]	0.00	0.00	-	0.01	1.22	[0.13, 12.50]	0.66	0.96	[0.83, 1.10]
	No	462	0.34			0.11			0.02			0.01			0.69		
Musculoskeletal/ rheumatological	Yes	114	0.30	0.96	[0.70, 1.30]	0.15	1.49	[0.90, 2.50]	0.02	1.61	[0.30, 9.09]	0.00	0.00	-	0.69	1.02	[0.89, 1.18]
	No	475	0.32			0.10			0.01			0.01			0.69		
Endocrinal	Yes	72	0.22	0.70	[0.45, 1.11]	0.08	0.75	[0.33, 1.69]	0.00	0.00	-	0.01	2.50	[0.28, 25.00]	0.56	0.80	[0.65, 0.99]
	No	517	0.33			0.11			0.01			0.01			0.71		
Other physical illness	Yes	307	0.29	0.86	[0.68, 1.10]	0.12	1.23	[0.77, 1.96]	0.01	0.36	[0.07, 1.82]	0.01	0.93	[0.13, 6.60]	0.67	0.96	[0.86, 1.06]
	No	282	0.34			0.10			0.02			0.01			0.71		
**No medical history**	Yes	145	0.39	**1.30***	[1.01, 1.67]	0.11	1.01	[0.57, 1.67]	0.02	2.38	[0.52, 11.11]	0.01	1.00	[0.10, 10.00]	0.74	1.09	[0.97, 1.22]
	No	444	0.29			0.11			0.01			0.01			0.67		
**Obstetric (current pregnancy)**																	
Multiparous	Yes	309	0.34	1.19	[0.95, 1.47]	0.11	0.94	[0.63, 1.41]	0.01	0.78	[0.24, 2.56]	0.01	0.63	[0.15, 2.63]	0.73	**1.16** **	[1.04, 1.27]
	No	422	0.31			0.13			0.02			0.02			0.66		
Unplanned/ unwanted pregnancy	Yes	174	0.21	0.58	[0.42, 0.80]	0.08	0.70	[0.39, 1.27]	0.01	0.52	[0.12, 2.13]	0.03	**11.99***	[1.33, 100.00]	0.62	0.87	[0.76, 0.99]
	No	347	0.34			0.11			0.02			0.003			0.69		
Complications during pregnancy	Yes	216	0.25	0.82	[0.55, 1.21]	0.07	0.71	[0.31, 1.60]	0.01	1.12	[0.13, 9.55]	0.02	1.54	[0.18, 13.30]	0.59	0.79	[0.67, 0.94]
	No	82	0.32			0.10			0.01			0.01			0.74		

Preterm delivery	Yes	61	0.26	0.95	[0.64, 1.73]	0.05	0.55	[0.16, 1.85]	0.02	2.38	[0.03, 6.60]	0.00	0.00	-	0.61	0.91	[0.72, 1.15]
	No	146	0.27			0.09			0.01			0.02			0.66		
Caesarean delivery	Yes	151	0.25	0.84	[0.58, 1.20]	0.11	1.40	[0.70, 2.82]	0.01	0.50	[0.05, 5.23]	0.01	0.50	[0.04, 5.63]	0.65	0.98	[0.84, 1.16]
	No	151	0.30			0.08			0.01			0.01			0.66		
Complications during childbirth	Yes	199	0.26	0.79	[0.53, 1.19]	0.10	1.74	[0.62, 4.85]	0.01	0.71	[0.08, 6.18]	0.01	0.70	[0.05, 9.57]	0.66	0.97	[0.81, 1.18]
	No	71	0.34			0.06			0.01			0.01			0.69		
**Obstetric (previous pregnancies, if applicable)**																	
Abortion	Yes	151	0.32	1.04	[0.73, 1.25]	0.13	1.16	[0.53, 1.38]	0.02	1.45	[0.18, 2.63]	0.01	0.55	[0.21, 15.27]	0.72	1.09	[0.96, 1.22]
	No	496	0.31			0.11			0.01			0.01			0.68		
Miscarriage	Yes	165	0.30	0.99	[0.77, 1.32]	0.10	0.88	[0.68, 1.87]	0.01	0.86	[0.26, 5.13]	0.00	0.00	-	0.65	0.96	[0.85, 1.10]
	No	481	0.31			0.12			0.01			0.01			0.69		
Complications during pregnancy[h]	Yes	142	0.30	0.93	[0.56, 1.54]	0.14	1.41	[0.51, 4.00]	0.01	0.27	[0.02, 4.17]	0.00	-	-	0.71	0.98	[0.79, 1.22]
	No	40	0.33			0.10			0.03			0.00			0.73		
Complications during childbirth[h]	Yes	81	0.32	1.23	[0.70, 2.17]	0.09	0.79	[0.27, 2.33]	0.01	-	-	0.00	-	-	0.74	1.22	[0.94, 1.59]
	No	46	0.26			0.11			0.00			0.00			0.61		

prop: proportion of women with a given outcome; **$_{a}$RR**: Adjusted relative risks, i.e. ratio of the proportion of women with a given outcome among those exposed over that proportion among those unexposed to the factor; **CI**: Confidence intervals.

[a]The psychiatric disorders newly diagnosed during postpartum refer to disorders that were not reported in the woman's past history, or a new psychiatric episode when a previous one was completely resolved before postpartum.

[b]Bipolar disorder included: type I, type II, atypical or unspecified, or cyclothymic disorder.

[c]SZ-related disorder included: schizophrenia, schizoaffective disorders, and substance-induced psychotic disorder.

[d]Any psychiatric disorder included the diagnosis of at least one psychiatric disorder (any one) diagnosed during postpartum.

[e]Due to missing data in medical records, the sum of the number of women having (or not) a given risk factor does not always add up to 772.

[f]A woman can contribute to the prevalence of more than one diagnosis. Hence, each diagnosis is treated as a dichotomous variable (presence vs absence) and consequently, the sum of the percentages is not interpretable and can exceed 100%.

[g]aRR is the adjusted Relative Risk, i.e., the risk of the incidence of a new specific disorder during postpartum among women having a given risk factor over that risk among women not having the risk factor, adjusted for the age of the mother at childbirth, based on a Poisson Regression model.

[h]These factors only apply to the multiparous women who have already had another child before this follow-up at the perinatal psychiatry clinic.

[I]This factor has not been adjusted for maternal age at childbirth.

* $p \leq 0.05$ ** $p \leq 0.01$ ***$p \leq 0.001$

Table S3

Significant factors associated with pregnancy-onset comorbidities[a] *including a SMI among the cohort of women who required psychiatric care in a perinatal psychiatry clinic and were followed up to pregnancy (n = 927). According to Poisson regression models that included one factor at a time and were adjusted for the mother's age at childbirth, an adjusted relative risk (aRR) was estimated for each factor.*

**Associated factors**[b]			**Pregnancy-onset comorbidities**					
			**Prenatal depression + anxiety disorder** n = 57			**Bipolar disorder + anxiety disorder** n = 19		
		**N**	**prop**	**$_a$RR**[c]	**95% CI**	**prop**	**$_a$RR**	**95% CI**
**Past psychiatric history**								
Postpartum psychosis[d]	Yes	5	0.00	0.00	-	0.20	**16.67****	[2.44, 100.00]
	No	307	0.08			0.01		
**No psychotropic medication**								
During pregnancy	Yes	213	0.14	**1.91***	[1.15, 3.23]	0.02	0.68	[0.24, 1.89]
	No	352	0.07			0.03		
**Sociodemographic**								
Nativity (Born outside of Canada)	Yes	90	0.11	**1.94***	[1.01, 3.70]	0.01	0.55	[0.07, 4.17]
	No	837	0.06			0.02		
**Medical history**								
Musculoskeletal/ rheumatological	Yes	143	0.11	**2.05***	[1.16, 3.57]	0.03	1.30	[0.43, 3.85]
	No	592	0.05			0.02		

prop: proportion of women with a given outcome; **$_a$RR**: Adjusted relative risks, i.e. ratio of the proportion of women with a given outcome among those exposed over that proportion among those unexposed to the factor; **CI**: Confidence intervals.

[a]The psychiatric comorbidities newly diagnosed during pregnancy refer to disorders that were both not reported in the woman's past history, or a new psychiatric episode when a previous one was completely resolved before pregnancy.

[b]Due to missing data in medical records, the sum of the number of women having (or not) a given risk factor does not always had up to 927.

[c]aRR is the adjusted Relative Risk, i.e. the risk of the incidence of specific comorbidities diagnosed during pregnancy among women having a given risk factor over that risk among women not having the risk factor, adjusted for the age of the mother at childbirth, based on a Poisson Regression model.

[d]These factors only apply to the multiparous women who have already had another child before this follow-up at the perinatal psychiatry clinic.

* $p \leq 0.05$ ** $p \leq 0.01$ ***$p \leq 0.001$

Table S4

Significant factors associated with postpartum-onset comorbidities[a] *including a SMI among the cohort of women who required psychiatric care in a perinatal psychiatry clinic and were followed up to postpartum (n = 772). According to Poisson regression models that included one factor at a time and were adjusted for the mother's age at childbirth, an adjusted relative risk (aRR) was estimated for each factor.*

Associated factors[b]			Postpartum-onset comorbidities: Postpartum depression + anxiety disorder n = 78			Postpartum depression + personality disorder n = 36			Postpartum depression + obsessive-compulsive disorder n = 32			Bipolar disorder + anxiety disorder n = 24			Bipolar disorder + personality disorder n = 17		
		**N**	**prop**	**$_{a}$RR**[c]	**95% CI**	**prop**	**$_{a}$RR**	**95% CI**	**prop**	**$_{a}$RR**	**95% CI**	**prop**	**$_{a}$RR**	**95% CI**	**prop**	**$_{a}$RR**	**95% CI**
**<u>Past psychiatric history</u>**																	
Depressive disorder	Yes	275	0.12	**1.69***	[1.04, 2.78]	0.06	1.49	[0.75, 2.94]	0.04	1.00	[0.47, 2.13]	0.05	**2.74***	[1.04, 7.14]	0.03	1.47	[0.52, 4.17]
(any one)	No	348	0.07			0.04			0.05			0.02			0.02		
Hypersensitivity to	Yes	47	0.21	**2.20****	[1.22, 4.00]	0.09	1.89	[0.68, 5.26]	0.15	**4.19*****	[1.92, 9.09]	0.04	1.35	[0.33, 5.56]	0.06	3.23	[0.94, 11.11]
hormone fluctuations	No	702	0.10			0.05			0.04			0.03			0.02		
(Hypo)/manic	Yes	14	0.07	0.70	[0.11, 4.55]	0.07	1.61	[0.24, 10.00]	0.00	*0.00*	-	0.21	**7.50*****	[2.50, 25.00]	0.14	**7.51****	[1.89, 33.33]
subclinical symptoms	No	735	0.10			0.05			0.04			0.03			0.02		
Anxiety subclinical	Yes	130	0.18	**2.03****	[1.30, 3.23]	0.05	1.22	[0.55, 2.70]	0.07	1.89	[0.88, 4.00]	0.04	1.25	[0.47, 3.33]	0.02	0.69	[0.16, 3.03]
symptoms	No	619	0.09			0.05			0.04			0.03			0.02		
Intrusive thoughts of	Yes	13	0.15	1.47	[0.39, 5.56]	0.00	0.00	-	0.08	1.89	[0.27, 12.50]	0.15	**4.14***	[1.06, 16.67]	0.08	2.56	[0.44, 14.28]
harming the infant[d]	No	290	0.11			0.06			0.04			0.04			0.03		
Cluster C personality	Yes	351	0.13	**1.86****	[1.20, 2.86]	0.04	0.81	[0.42, 1.54]	0.04	1.08	[0.54, 2.13]	0.04	2.00	[0.88, 4.55]	0.02	0.71	[0.26, 1.92]
traits	No	421	0.07			0.05			0.04			0.02			0.03		
Somatic related	Yes	6	0.33	**3.97***	[1.30, 12.50]	0.17	4.17	[0.66, 25.00]	0.33	**9.02*****	[2.86, 25.00]	0.00	0.00	-	0.00	0.00	-
disorder	No	617	0.09			0.05			0.04			0.03			0.02		
**Suicidal behavior**	Yes	74	0.05	0.46	[0.17, 1.23]	0.09	1.89	[0.84, 4.35]	0.03	0.58	[0.15, 2.27]	0.07	2.44	[0.97, 6.25]	0.08	**4.16****	[1.49, 14.29]
	No	675	0.11			0.04			0.04			0.03			0.02		
**<u>Pregnancy-onset psychiatric disorders</u>**																	
Intrusive thoughts of	Yes	18	0.00	0.00	-	0.11	**12.91****	[1.89, 100.00]	0.06	-	-	0.00	0.00	-	0.00	0.00	-
harming the infant	No	345	0.01			0.01			0.00			0.01			0.003		
**No new pregnancy**	Yes	433	0.17	**10.26*****	[4.17, 25.00]	0.07	**4.07****	[1.61, 10.00]	0.07	**22.16****	[1.13, 100.00]	0.04	**2.76***	[1.01, 7.69]	0.03	2.94	[0.84, 10.00]
**onset disorder**	No	307	0.02			0.02			0.003			0.02			0.01		
**<u>No psychotropic medication</u>**																	
During pregnancy	Yes	166	0.07	**2.41***	[1.01, 5.88]	0.04	2.44	[0.69, 8.33]	0.02	3.23	[0.60, 16.67]	0.01	0.20	[0.03, 1.59]	0.01	0.41	[0.05, 3.70]
	No	268	0.03			0.01			0.01			0.03			0.01		
**<u>Sociodemographic</u>**																	
Nativity (Born outside	Yes	82	0.16	**1.75***	[1.01, 3.03]	0.05	1.15	[0.42, 3.13]	0.05	1.23	[0.44, 3.45]	0.01	0.36	[0.05, 2.56]	0.00	0.00	-
of Canada)	No	690	0.09			0.05			0.04			0.03			0.02		

prop: proportion of women with a given outcome; **$_{a}$RR**: Adjusted relative risks, i.e. ratio of the proportion of women with a given outcome among those exposed over that proportion among those unexposed to the factor; **CI**: Confidence intervals.

[a]The psychiatric comorbidities newly diagnosed during postpartum refer to disorders that were both not reported in the woman's past history, or a new psychiatric episode when a previous one was completely resolved before postpartum.

[b]Due to missing data in medical records, the sum of the number of women having (or not) a given risk factor does not always had up to 772.

[c]aRR is the adjusted Relative Risk, i.e. the risk of the incidence of specific comorbidities diagnosed during postpartum among women having a given risk factor over that risk among women not having the risk factor, adjusted for the age of the mother at childbirth, based on a Poisson Regression model.

[d]These factors only apply to the multiparous women who have already had another child before this follow-up at the perinatal psychiatry clinic.

* $p \leq 0.05$ ** $p \leq 0.01$ ***$p \leq 0.001$
